# Genome‐wide association mapping for field and seedling resistance to the emerging *Puccinia graminis* f. sp. *tritici* race TTRTF in wheat

**DOI:** 10.1002/tpg2.20274

**Published:** 2022-10-20

**Authors:** Tamrat Negash, Erena Aka Edae, Lidiya Tilahun, James A. Anderson, Matthew N. Rouse, Prabin Bajgain

**Affiliations:** ^1^ Ethiopian Institute of Agricultural Research Kulumsa Ethiopia; ^2^ Dep. of Plant Pathology Univ. of Minnesota St. Paul MN USA; ^3^ Dep. of Agronomy and Plant Genetics Univ. of Minnesota St. Paul MN USA; ^4^ USDA–ARS Cereal Disease Laboratory St. Paul MN 55108 USA

## Abstract

Stem rust of wheat (*Triticum* spp.), caused by *Puccinia graminis* f. sp. *tritici* (*Pgt*), is one of the most impactful wheat diseases because of its threat to global wheat production. While disease mitigation has primarily been achieved through the deployment of resistant wheat varieties, emerging new virulent races continue to pose risks to the crop. For example, races such as Ug99 (TTKSK), TKTTF, and TTRTF have caused epidemics in different wheat growing regions of the world in recent years. A continual search for new and effective sources of resistance is therefore necessary to safeguard wheat production. This study assessed a breeding panel from the Ethiopian Institute of Agricultural Research (EIAR) wheat breeding program for seedling and field plant resistance to TTRTF and reports genomic regions conferring resistance to TTRTF. Trait correlations (*r*) were medium to strong (range = .38–.71) and heritabilities were moderate (.32–.56). Association analysis for resistance to TTRTF resulted in detection of 20 markers in 11 chromosomes; the marker *S1B_175439851* was associated with resistance at both seedling and adult plant stages. Models with two to four QTL combinations reduced seedling and field disease severity by 12–48 and 9–17%, respectively. Genomic prediction for TTRTF resistance resulted in low to moderately‐high predictions (mean correlations of .25–.47). Identification of resistant lines and QTL in the EIAR population is expected to assist in selection toward improved resistance to TTRTF. Specifically, the application of genomic selection (GS) in identifying resistant lines in future related breeding populations will further assist breeding efforts against this new stem rust pathogen race.

## Core Ideas

- Stem rust threatens wheat production in all wheat growing regions of the world.
- Continual search for resistance genes is needed to safeguard wheat from new races such as TTRTF.
- QTL associated with TTRTF resistance can reduce stem rust severity by up to 48% when deployed in combinations.
- By predicting resistance in future populations, GS could aid in selection of resistant lines.

**Abbreviations**
BLUE: best linear unbiased estimate
COI: coefficient of infection
EIAR: Ethiopian Institute of Agricultural Research
GS: genomic selection
GWAS: genome‐wide association study
LD: linkage disequilibrium
PC: principal component
QTL: quantitative trait loci
SNP: single‐nucleotide polymorphism

## 1 INTRODUCTION

Wheat (*Triticum* spp) is the most widely cultivated crop in the world (https://www.fao.org/faostat/en/#home) and accounts for ∼20% of calories and protein consumed by humans globally (Shiferaw et al., 2013). In North Africa and Western and Central Asia, wheat is even more significant, as it accounts for 39–47% of calories consumed (Shiferaw et al., 2013). Two species account for nearly all of wheat production globally: 95% common wheat (*T. aestivum* L.) and 5% durum wheat [*T. turgidum* L. subsp. *durum* (Desf.) van Slageren] (Shewry, 2009). Wheat stem rust, caused by *Puccinia graminis* f. sp. *tritici* (*Pgt*), reduces wheat yields and was ranked at the 11th most impactful disease of wheat globally, though, in sub‐Saharan Africa, it was ranked as the most significant disease of wheat (Savary et al., 2019). However, before stem‐rust‐resistant varieties of wheat were deployed around the world, primarily in the 1960s, stem rust was regarded as the most devastating and feared wheat disease (Van der Plank, 1963; Roelfs, 1985).

Though resistant varieties have largely mitigated stem rust damage to wheat in recent decades, new virulent strains of *Pgt*, such as Ug99, have emerged and spread throughout eastern and southern Africa and the Middle East (Pretorius et al., 2000). Ug99 presented a unique problem since 80–94% of wheat breeding lines and cultivars from various germplasm groups around the world were characterized as susceptible (Singh et al., 2006). Ug99 was described as race TTKSK (Jin et al., 2008) and numerous variants of TTKSK have been described with virulence to additional stem rust resistance genes including *Sr24*, *Sr36*, and *SrTmp* (Jin et al., 2009; Newcomb et al., 2016). In addition to Ug99, races such as TKTTF emerged as an epidemic‐causing strain in Ethiopia in 2013 (Olivera et al., 2015) and race TTRTF caused a stem rust epidemic in Sicily in 2016 (Bhattacharya, 2017; Patpour et al., 2018). Race TTRTF was first detected in Georgia in 2014 (Olivera et al., 2019) but has subsequently spread into Egypt, Eritrea, Ethiopia, Hungary, Italy, and Iran (Esmail & Szabo, 2018; Patpour et al., 2020; Tesfaye et al., 2020). Race TTRTF is virulent to several wheat stem rust resistance genes that are effective to Ug99 including *Sr37*, *Sr50*, and *Sr13b* (Zhang et al., 2017; Olivera et al., 2019). Phylogenetic analysis using single‐nucleotide polymorphism (SNP) marker data indicated that race TTRTF belonged to *Pgt* clade III‐B, closely related to clade III‐A (race TRTTF, first detected in Yemen in 2006 [Olivera et al., 2012]) but only distantly related to other races such as TTKSK (Ug99) or TKTTF (Olivera et al., 2019).

**Core Ideas**

- Stem rust threatens wheat production in all wheat growing regions of the world.
- Continual search for resistance genes is needed to safeguard wheat from new races such as TTRTF.
- QTL associated with TTRTF resistance can reduce stem rust severity by up to 48% when deployed in combinations.
- By predicting resistance in future populations, GS could aid in selection of resistant lines.

Though race TTRTF caused an epidemic on durum wheat in Sicily in 2016 (Patpour et al., 2020), remarkably little research has subsequently been reported on wheat susceptibility or resistance to this potentially threatening, highly virulent race. Edae and Rouse (2020) assessed a panel of lines representing common spring wheat in North America and found that 80.2% were susceptible as seedlings to race TTRTF. A study of the CIMMYT durum wheat breeding germplasm indicated that 43.6% were susceptible as seedlings to race TTRTF (Megerssa et al., 2021). Although seedling studies are useful for identifying wheat lines that possess effective major resistance genes, field experiments are needed to accurately assess the response of wheat lines in the conditions in which they would be exposed to the disease. Furthermore, though field experiments are often conducted using a bulk inoculum of multiple virulent *Pgt* races (Olivera et al., 2012; Rouse et al., 2014), field response to a specific *Pgt* race is most accurately determined in field experiments inoculated with a single *Pgt* race. Field screening of North American spring wheat breeding lines found that field responses to different *Pgt* races as assessed in single‐race nurseries could actually be significantly negatively correlated, indicating substantial race specificity (Edae et al., 2018). Indeed, this result was validated through association mapping of different genomic regions effective in the field to the different races. Similarly, assessment of common and durum wheat breeding lines adapted to Ethiopia in single‐race nurseries identified consistent race‐specific responses in lines that were otherwise susceptible as seedlings to particular *Pgt* races (Hundie et al., 2019). However, association mapping of Ethiopian durum wheat response to *Pgt* races JRCQC and TKTTF in single‐race nurseries revealed the same genomic regions effective to each race (Megerssa et al., 2021). To date, no study has reported the field response of wheat lines to race TTRTF.

Associations between genomic markers and plant response to stem rust have been carried out in several studies using interval mapping and single‐marker analysis (Singh et al., 2015). Single‐marker analysis, typically done as association mapping or genome‐wide association study (GWAS), identifies significant marker–trait associations by exploiting historical recombination events and linkage disequilibrium (LD) existent in the population (Weir, 2008). As LD, allele frequencies, and population structure can affect GWAS models (Weir, 2008; Sul et al., 2018), it is important to use abundant markers free of ascertainment bias while controlling for underlying population structure (Heslot et al., 2013; Sul et al., 2018). Single‐nucleotide polymorphism markers are primarily discovered via the application of high‐throughput sequencing platforms. Because of their abundance, SNPs more accurately represent genome‐wide LD and allelic diversity while significantly reducing ascertainment bias thereby aiding in accurate identification of significant loci during a GWAS (Heslot et al., 2013; Alqudah et al., 2020). Markers discovered by GWAS have been successfully validated for use in marker‐assisted breeding to improve resistance to stem rust (Yu et al., 2017; Mourad et al., 2018). Another widely used approach in recent years to improve the frequency of alleles conferring resistance to stem rust in wheat breeding populations is genomic selection (GS) (Haile et al., 2020). An in vivo marker‐based selection approach, GS trains statistical models from phenotypic and genotypic data and applies the model on a genotyped population to predict trait performance. A GS model can be effectively used to select genotypes for a trait that is controlled by many small‐ or medium‐effect loci (Lorenz et al., 2011). Therefore, if effective, GS can aid in selection of wheat lines with increased resistance to TTRTF in future breeding populations.

This study was therefore carried out with the primary goals of evaluating the seedling and field response of wheat lines to race TTRTF and discovering genetic markers in significant association with resistance to this race. For this purpose, we screened advanced spring common wheat breeding germplasm from the Ethiopian Institute of Agricultural Research (EIAR) wheat breeding program for resistance to TTRTF. The population was evaluated for disease response, trait correlations, and heritabilities as well as LD and population structure. A GWAS was performed to uncover genomic regions controlling TTRTF resistance. The usefulness of GS in selecting resistant wheat lines was also evaluated by using a genomic prediction model to predict disease response.

## 2 MATERIALS AND METHODS

### 2.1 Wheat germplasm

A total of 183 common spring wheat breeding lines and cultivars representative of the spring wheat breeding program of the EIAR were evaluated in this study. The 183 wheat lines included 30 cultivars and 153 advanced breeding lines. In the field studies, five check cultivars were also included: ‘Arendeto’, ‘Danda'a’, ‘Digalu’, ‘Kingbird’, and ‘Kubsa’ (Hundie et al., 2019). Arendeto is an Ethiopian durum wheat cultivar, and the other checks are Ethiopian common spring wheat cultivars.

### 2.2 TTRTF single‐race field nursery

During the ‘main’ and ‘off’ wheat growing seasons in Ethiopia during the months of June through October 2018 and February through May 2019, respectively, we assessed the 183 wheat lines for response to *Pgt* race TTRTF in single‐race nurseries (Hundie et al., 2019). The single‐race nurseries were established at the EIAR Kulumsa Agricultural Research Center and planted a minimum of 100 m away from other wheat plots. Briefly, the 183 wheat lines were sown in single 1‐m rows planted perpendicular to spreader rows of the wheat lines Kubsa and LMPG‐6. The five check cultivars were repeated in a randomized augmented design with five replications. At the stem elongation stage, a 2 mg L^−1^ suspension of urediniospores creating cocacola color in Soltrol 70 (a lightweight mineral oil, ConocoPhillips Inc.) was sprayed onto the spreader rows using an Ulva+ sprayer (Micron Sprayers). The *Pgt* urediniospores were of race TTRTF collected at the Ambo Agricultural Research Center. Starting at the milky‐ripe growing stage through the soft‐dough stage, the wheat lines were evaluated three times in each season for stem rust disease severity on a 0‐to‐100% modified Cobb scale (Peterson et al., 1948) and infection response. Infection responses were recorded as resistant (R), moderately resistant (MR), moderately susceptible (MS), and susceptible (S) (Roelfs et al., 1992). When multiple infection responses were observed on the same plot, all infection responses were recorded.

### 2.3 Seedling phenotyping

The 183 wheat lines were evaluated in two separate experiments for seedling response to *Pgt* race TTRTF (received from the Ambo Agricultural Research Center) at the EIAR Kulumsa Agricultural Research Center (Woldeab et al., 2019). Briefly, five wheat lines (five seed per line) were sown in clusters in plastic pots containing a 2:1:1 ratio of sterilized soil/sand/farmyard manure. Approximately 8 d after planting, seedlings were inoculated with a 14 mg 0.75 ml^−1^ urediniospore–Soltrol 70 suspension by connecting a single gelatin capsule to a pressurized atomizer (30 kPa), where one capsule was used to inoculate 12 pots total. After inoculation, plants were placed in a dew chamber covered by an opaque polyethylene sheet to maintain darkness where humidity was maintained by ultrasonic humidifiers overnight (16 h total). The following morning, the plants were exposed to natural light inside the same humid chambers for 4 h before turning off the humidifiers and allowing the plants to dry. Plants were then placed in a greenhouse for 14 d before assessing seedling infection types on a 0‐to‐4 scale (Stakman et al., 1962). Infection types 0–2 were considered as resistant and 3–4 as susceptible.

### 2.4 Statistical analysis of trait data

Infection responses recorded in the field nurseries were converted to a 0‐to‐1 linear scale according to Gao et al. (2016). Furthermore 0‐to‐100 disease severity values were multiplied by the 0–1 linear infection responses to obtain the coefficient of infection (COI) values for each plot. Seedling infection types were converted to a 0‐to‐9 linear scale following Gao et al. (2016). Both COI and linear seedling scores were assessed using a mixed model in the R package sommer (v4.1.6) to correct for trial effect, that is, environmental and replication variability. The fixed effects of trial and replication variables estimated by the mixed model were removed from trait values of each line to obtain the best linear unbiased estimate (BLUE) for each line. Replicates were then averaged in all trials and all subsequent analyses used mean BLUEs. Broad‐sense trait heritability (*H* ^2^) was estimated on a line‐mean basis (Falconer et al., 1996) using the function ‘h2.fun’ in sommer.

### 2.5 Genotyping

DNA was extracted from the wheat lines using a modified SDS DNA extraction protocol (Kosgey et al., 2021) from 10–15 cm long leaf tissue. Extracted DNA was normalized to 20 ng μl^−1^ and double digested with the enzymes *Msp*I and *Pst*I (Poland et al., 2012). Sequencing libraries were prepared by pooling samples at 192‐plex and sequenced on Illumina's HiSeq 2500. Reads obtained were filtered for a minimum quality score (*Q*) ≥ 30 and demultiplexed using ‘Sabre’ (https://github.com/najoshi/sabre). Reads for each line in the population were first aligned to the IWGSC reference genome v1.0 (Appels et al., 2018) using ‘bwa mem’ (Li, 2013) followed by variant detection using ‘samtools mpileup’ and ‘bcftools’ (Li et al., 2009; Li, 2011). Variants (SNPs, primarily) with minor allele frequency <3% and >20% missing data were discarded resulting in 5,181 high‐quality SNPs retained for downstream analyses. This set of 5,181 SNPs was imputed using the LD‐kNNi method (Money et al., 2015) in Tassel 5.2.74 (Bradbury et al., 2007) with default parameters, that is, 30 high‐LD sites and 10 nearest neighbors. Imputation accuracy of the imputed dataset was calculated by masking known genotypes of 20% alleles in the input file prior to imputation and comparing the predicted alleles with the masked genotypes. In case of unresolved missing genotypes postimputation, no forced imputation was carried out. After discarding lines with poor genotypic information, 178 of the 183 lines were used in association analysis.

The population was also screened for the presence of the 1BL.1RS wheat–rye (*Secale cereale* L.) translocation, as the translocated segment is known to possess the stem rust resistance gene *Sr31* (McIntosh et al., 1995). For this purpose, three molecular markers linked with the translocation were used: *SCM9*, *1A1R_8035*, and *1B1R_6011*. Presence of *SCM9* (Kofler et al., 2008) was assessed on a 1% agarose gel, whereas the other two markers are Kompetitive allele‐specific polymerase chain reaction assays described in Ward et al. (2019) and assayed according to Nirmala et al. (2017).

### 2.6 Linkage disequilibrium and population structure

Estimates of pairwise LD among the SNP markers were obtained from Tassel 5.2.74 with a sliding window of 50 SNP markers. The LD (*r* ^2^) values were plotted against the physical genomic distance (mega base pairs, Mbp) of the IWGSC v1.0 reference genome. A locally weighted polynomial regression (LOWESS) curve was fitted to the distribution to track the pattern of LD decay. The LD decay distance was calculated using Hill and Weir's method (1988) and assessed at the conventionally accepted *r* ^2^ value of .2 (Vos et al., 2017). Genetic relationship among the lines in the population is discussed as kinship among the lines and was estimated in Tassel 5.2.74 using the Centered_IBS method using default parameters, that is, maximum number of alleles = 6. Presence of population structure and stratification was assessed using principal component (PC) analysis with the function ‘prcomp’ in R. Subpopulations observed during the PC analysis were visualized by grouping the lines using a *k*‐means clustering algorithm (Adeyemo et al., 2020).

### 2.7 GWAS and genomic selection

Significant associations between SNP markers and stem rust phenotypic data were carried out using the ‘GAPIT’ package in R v4.0.2 (Lipka et al., 2012). The three *Sr31* markers (*SCM9*, *1A1R_8035*, and *1B1R_6011*) were included in the GWAS by assigning them arbitrary base pair positions on an unknown or unmarked chromosome. Three PC values were included in the GWAS model to control for population structure as recommended by the results of the ‘Model.selection = TRUE’ option. The inclusion of kinship matrix was not found to significantly change mapping results and thus was not used in the GWAS model. Quantitative trait loci (QTL) were declared significant at a logarithm of the odds threshold of 3.0 or higher. A search for candidate genes surrounding the discovered QTL regions was done spanning 2 Mbp of the identified QTL, that is, 1 Mbp to the left and 1 Mbp to the right of the significant SNP markers. Search was limited to IWGSC v1.0 high‐confidence genes that were annotated with functional characterization for several classes related to defense mechanisms as proposed by Kourelis and van der Hoorn (2018) and Jan et al. (2021).

The usefulness of genomic prediction models in predicting TTRTF resistance in this population was evaluated using the R package ‘rrBLUP’ (Endelman, 2011). A fourfold cross‐validation scheme was implemented to obtain trait predictive abilities, that is, correlations (*r*) between adjusted trait values (BLUEs) and model‐predicted trait values. In the fourfold cross‐validation method, 75% of the population was used as the training set to predict the validation set, which comprises the remaining 25%. Both training and validation sets were randomly sampled without replacement.

## 3 RESULTS

### 3.1 Disease phenotypic data distribution and properties

**FIGURE 1:**
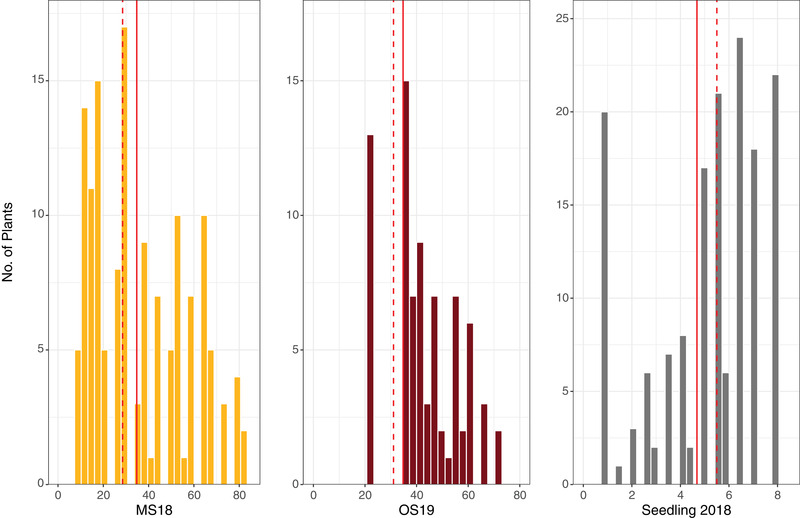
Histograms of disease severity observed during 2018 main season (MS18), 2019 off season (OS19), and data obtained from seedling assays (Seedling 2018). Seedling data are shown in linear scale (0–9). Solid and dashed red lines indicate mean and median values of the distribution, respectively

The population exhibited a broad‐spectrum of responses to TTRTF during both field and seedling screening (Figure 1). The COIs for field response in both field trials were nearly the same with an average COI of 34.4 and 34.6 in MS18 and OS19, respectively. The highest susceptibility was observed in MS18 (88.5). Seedling assays revealed ∼44% of the population being resistant to TTRTF. On a linear scale of 0 to 9, the mean response was 4.5 (median = 5.5) and ranged from 0 to 8.5. Correlations (*r*) between the three data sets showed medium to strong relationships with the strongest correlation observed between the two Ethiopian field trials (*r* = .71). Correlation values between seedling assay and MS18 and OS19 were .38 and .49, respectively. Trait heritability estimates were the highest in 2018 main season nursery (*H* ^2^ = .56) followed by seedling assay (.40) and 2019 off season nursery (.32).

We identified 20 lines with resistance effective in both field seasons (COI levels of 25 or below). A total of 19 of these lines were also resistant as seedlings to race TTRTF, indicative of all‐stage resistance to TTRTF. One such line, cultivar Hulluka was previously characterized to possess *Sr24* (Hundie et al., 2019). Cultivar K6295‐4A was susceptible as a seedling to race TTRTF with infection type ‘3’ in addition to exhibiting COI levels <25 in both seasons. Therefore, K6295‐4A was identified as possessing adult plant resistance to race TTRTF. We also identified an additional four lines with average COI levels <25 but where COI reached as high as 33 in the 2019 offseason: ETBW9087, ETBW9549, ETBW9555, and ETBW9636. These four lines were also susceptible as seedlings, indicative of adult plant resistance effective to race TTRTF.

### 3.2 Population structure and linkage disequilibrium

**FIGURE 2:**
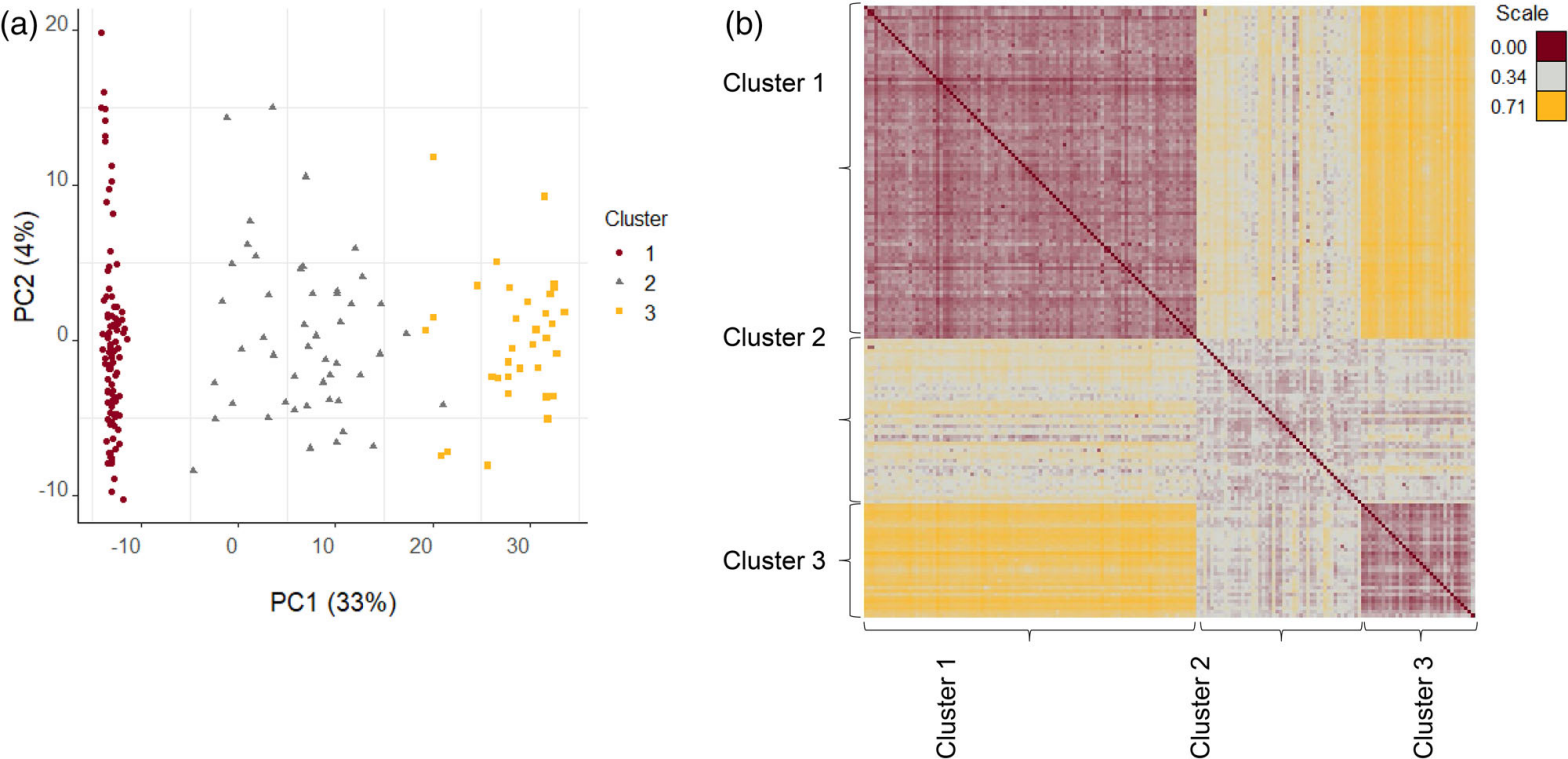
Population structure and relationship among the lines used in this study. The genetic distance among lines based on principal component (PC) analysis shown in (a) and (b) displays a heatmap based on additive genetic relationship. In both panels, lines were grouped using a *k*‐means clustering method

A strong population structure was observed among the lines used in this study (Figure 2). Principal component analysis revealed that the first axis alone explained 33% of the genetic variation in the population. Grouping of lines based on the *k*‐means clustering method indicated the presence of three subgroups and is visualized in Figure 2a. The three‐cluster subpopulation structure was corroborated by strong genetic relationships observed among the lines in each subgroup (Figure 2b).

Linkage disequilibrium extended the longest in the D genome with half‐decay distance of 16.7 Mbp at *r^2^* = .20 (Supplemental Figure S1). For A and B genomes, this value was 2.5 and 3.7 Mbp, respectively. Additionally, the average *r^2^* value was also found to be the largest in the D genome (.23) followed by the A and B genomes (.16 in both).

### 3.3 Marker–trait associations

**FIGURE 3:**
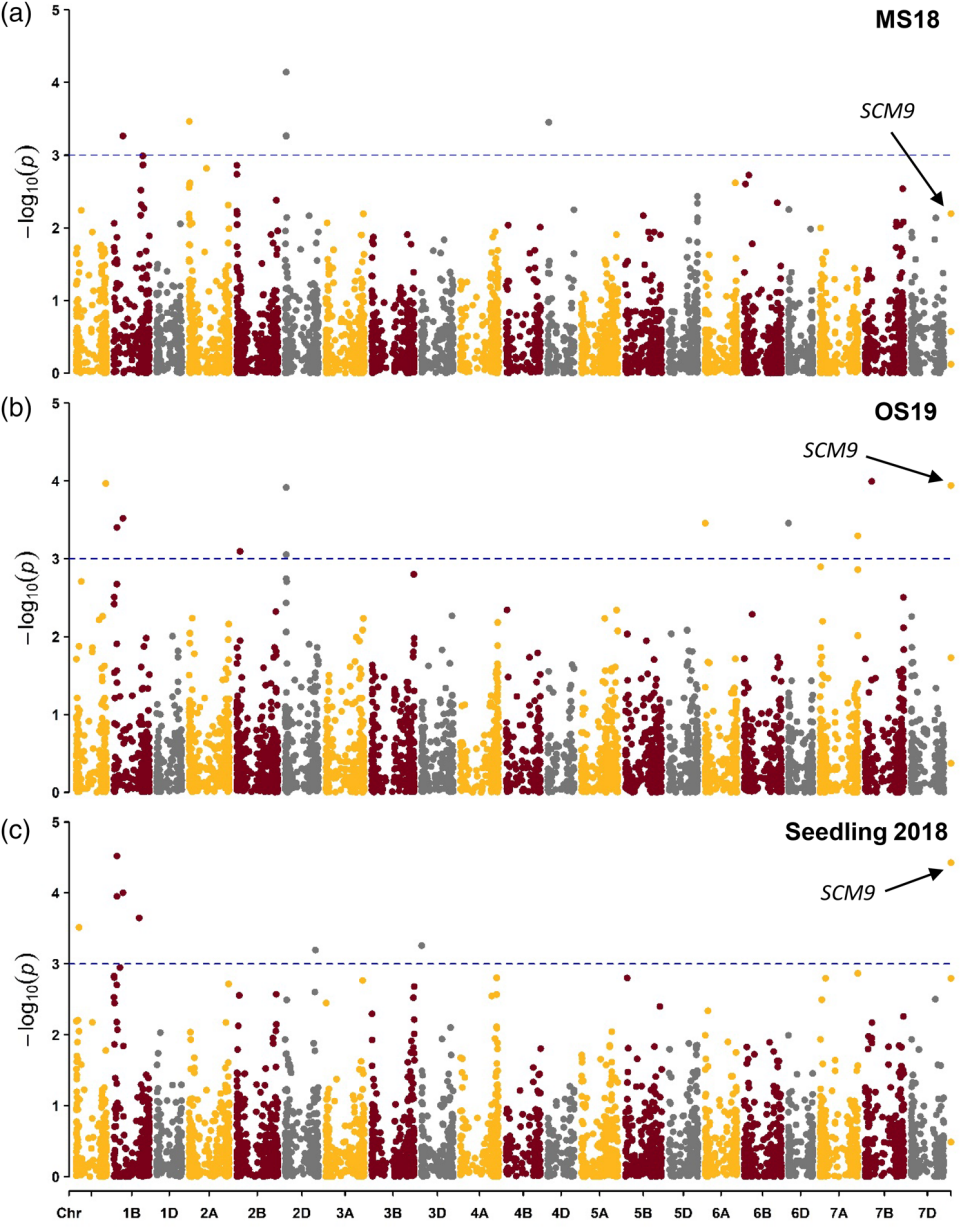
Manhattan plots showing association statistics of genome‐wide single‐nucleotide polymorphism markers with the three stem rust data sets: (a) 2018 main season (MS18), (b) 2019 off season (OS19), and (c) the seedling assays (Seedling 2018). The marker *SCM9*, indicated by arrows, is associated with *Sr31* presence. The dashed blue line shown at *P* value ≥ 1 ×10^−3^ (i.e., logarithm of odds (LOD) value ≥ 3.0) was the significance threshold

A search for markers significantly associated with field and seedling resistance to TTRTF resulted in detection of 20 markers (19 SNPs and one *Sr31* marker) in 11 chromosomes (Table 1, Figure 3). Chromosome 2D had the largest number of significant markers (five). Seven markers were in significant association with TTRTF resistance in MS18, 11 in OS19, and eight with seedling resistance. The largest proportion of observed phenotypic variance (*R* ^2^) was explained by the marker *S1B_57131236* for the seedling assay with *R* ^2^ = 9.9%; the largest *R* ^2^ explaining the field data was 7.6% by marker *S7B_125884505* and was observed in OS19. The average number of favorable alleles in the population was 15 and ranged from seven to 21 with the lines BWCM270 and ETBW9313 having the highest number of favorable alleles (Supplemental Table S1). The 19 significant SNP markers were positioned within 2 Mbp of 519 candidate genes, of which, 103 belonged to 47 gene classes that are proposed to be involved in plant defense mechanisms (Supplemental Table S2).

**TABLE 1:** Single‐nucleotide polymorphism (SNP) markers significantly associated with the three stem rust data sets: 2018 main season (MS18), 2019 off season (OS19), and data obtained from the seedling assays (Seedling 2018)

					MS18			OS19			Seedling 2018		
SNP	Chr	Position	Alleles	MAF	*P* value	*R* ^2^	Allelic effect	*P* value	*R* ^2^	Allelic effect	*P* value	*R* ^2^	Allelic effect
		Mbp											
S1A_57399833	1A	57.4	C/T	0.09	4.10 × 10^−2^	1.64	−4.91	1.32 × 10^−2^	2.98	−3.78	3.05 × 10^−4^	7.35	−1.45
S1A_575239272	1A	575.24	T/G	0.11	4.89 × 10^−2^	1.52	−4.08	1.08 × 10^−4^	7.48	−5.14	1.66 × 10^−2^	3.17	−0.8
S1B_57131231	1B	57.13	C/G	0.12	1.35 × 10^−2^	2.4	−5.95	3.96 × 10^−4^	6.22	−5.4	1.12 × 10^−4^	8.46	−1.5
S1B_57131236	1B	57.13	T/A	0.11	3.14 × 10^−2^	1.82	4.92	2.12 × 10^−3^	4.64	4.46	3.01 × 10^−5^	9.94	1.56
S1B_175439851	1B	175.44	T/G	0.16	5.44 × 10^−4^	3.06	5.63	3.02 × 10^−4^	4.31	3.75	9.93 × 10^−5^	8.6	1.29
S1B_489545605	1B	489.55	G/T	0.12	7.61 × 10^−1^	0.04	−0.78	1.27 × 10^−1^	1.12	−2.48	2.25 × 10^−4^	7.69	−1.5
S2A_4620729	2A	4.62	C/G	0.41	3.42 × 10^−4^	5.15	−9	6.91 × 10^−2^	1.59	−2.74	2.30 × 10^−1^	0.79	‐0.44
S2B_67087867	2B	67.09	G/T	0.14	4.64 × 10^−2^	1.55	3.77	8.04 × 10^−4^	5.54	4.13	3.95 × 10^−1^	0.39	0.27
S2D_22816678	2D	22.82	A/C	0.47	5.38 × 10^−4^	4.8	9.71	1.21 × 10^−4^	7.37	6.92	1.80 × 10^−1^	0.98	0.62
S2D_22816689	2D	22.82	G/A	0.45	5.47 × 10^−4^	4.79	6.87	8.84 × 10^−4^	5.45	4.2	3.93 × 10^−1^	0.4	0.28
S2D_22816672	2D	22.82	G/C	0.44	7.20 × 10^−5^	6.39	7.87	1.81 × 10^−3^	4.78	3.89	2.91 × 10^−1^	0.61	0.34
S2D_22816702	2D	22.82	G/A	0.44	7.20 × 10^−5^	6.39	7.87	1.81 × 10^−3^	4.78	3.89	2.91 × 10^−1^	0.61	0.34
S2D_591388465	2D	591.39	C/T	0.16	9.47 × 10^−1^	0	0.17	7.86 × 10^−1^	0.04	0.44	6.38 × 10^−4^	6.55	−1.47
S3D_931530	3D	0.93	C/A	0.22	6.53 × 10^−1^	0.08	0.73	3.47 × 10^−1^	0.42	0.97	5.52 × 10^−4^	6.7	0.93
S4D_18464711	4D	18.46	G/C	0.38	3.52 × 10^−4^	5.13	−19.64	2.78 × 10^−2^	2.34	−7.44	1.95 × 10^−1^	0.91	−1.13
S6A_1757444	6A	1.76	A/G	0.26	1.51 × 10^−1^	0.8	2.31	3.50 × 10^−4^	6.34	3.68	1.01 × 10^−2^	3.66	0.67
S6D_2750348	6D	2.75	A/G	0.26	1.51 × 10^−1^	0.8	2.31	3.50 × 10^−4^	6.34	3.68	1.01 × 10^−2^	3.66	0.67
S7A_733820033	7A	733.82	G/A	0.16	2.71 × 10^−1^	0.47	−2.2	5.07 × 10^−4^	5.98	−4.44	1.36 × 10^−3^	5.74	−1.04
S7B_125884505	7B	125.88	A/C	0.13	4.89 × 10^−2^	1.52	3.95	1.01 × 10^−4^	7.55	5	1.11 × 10^−2^	3.56	0.83
Sr31_SCM9	NA	0	NA	0.16	6.39 × 10^−3^	2.94	5.69	1.15 × 10^−4^	7.42	5.01	3.73 × 10^−5^	9.7	1.34

*Note*. *R* ^2^ is the percentage of phenotypic variation explained by the marker. MAF, minor allele frequency

Few markers were significant among the different data sets:

1. Marker S1B_175439851 was significant in all three data sets.
2. Markers S2D_22816678 and S2D_22816689 were significant in MS18 and OS19.
3. Marker S1B_57131231 and SCM9 were significant in OS19 and the seedling assay.

### 3.4 QTL models and genomic selection for disease reduction

Quantitative trait loci with the highest amount of phenotypic variation explained (*R* ^2^) in each data set were evaluated in two to four QTL combinations to estimate their combined effects on disease severity. These combination models reduced field stem rust severity by 9−13% and seedling infection by 12−48% (Table 2). Overall, two‐QTL models were more successful in reducing disease levels in both field and seedling data sets compared with three‐ or four‐QTL models. Surprisingly, one combination of two QTL (*S2D_22816672* + *S7B_125884505*) showed a 10.5% reduction in field severity but 5.6% increase in severity at the seedling stage.

**TABLE 2:** Reduction in field and seedling stem rust disease severity from quantitative trait loci (QTL) combination models. Field severity scores are average values between MS18 (main season 2018) and OS19 (off season 2019)

QTL combinations	Field stem rust severity			Seedling linear score (0–9)		
	No QTL	With QTL	Reduction	No QTL	With QTL	Reduction
	%					%
S1B_57131236 + S1B_175439851	35.2 (0.2)	31.1 (1.2)	11.5	2.6 (0.2)	5.0 (0.1)	47.7
S1B_57131236 + S2D_22816672	35.9 (0.9)	31.2 (1.2)	13.0	3.6 (0.8)	4.8 (0.1)	25.3
S1B_57131236 + S7B_125884505	36.4 (1.3)	32.2 (2.1)	11.4	3.8 (1.0)	4.4 (0.5)	14.9
S1B_175439851 + S2D_22816672	36.0 (0.8)	32.2 (0.7)	10.5	3.4 (1.0)	4.9 (0.2)	29.9
S1B_175439851 + S7B_125884505	36.5 (1.2)	33.2 (1.2)	9.0	3.6 (1.2)	4.5 (0.6)	20.1
S2D_22816672 + S7B_125884505	37.2 (0.6)	33.3 (1.1)	10.5	4.6 (0.2)	4.4 (0.4)	−5.7
S1B_57131236 + S2D_22816672 + S7B_125884505	36.5 (1.1)	32.3 (1.8)	11.6	4.0 (0.9)	4.5 (0.4)	12.0
S1B_175439851 + S2D_22816672 + S7B_125884505	36.5 (1.0)	32.9 (1.2)	10.0	3.9 (1.0)	4.6 (0.5)	15.4
S1B_57131236 + S1B_175439851 + S7B_125884505	36.0 (1.2)	32.2 (1.8)	10.6	3.3 (1.0)	4.6 (0.5)	28.3
S1B_57131236 + S1B_175439851 + S2D_22816672 + S7B_125884505	36.2 (1.1)	32.2 (1.6)	11.0	3.6 (1.0)	4.7 (0.4)	22.8

*Note*. Values in parentheses are standard deviations.

**FIGURE 4:**
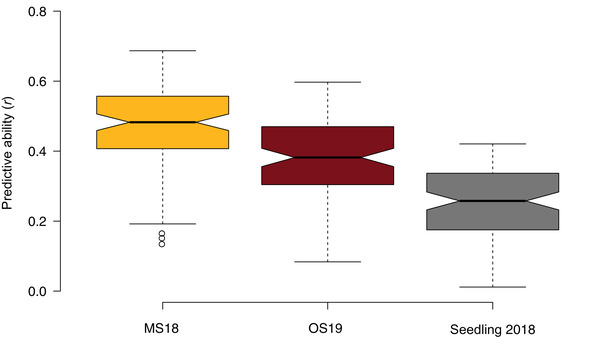
Predictive ability values for the three TTRTF data sets: MS18 is main season 2018, OS19 is off season 2019, and Seedling 2018 is data obtained from seedling assays

The suitability of GS in predicting rust response in each dataset was carried out using rrBLUP's ridge regression model and results are displayed in Figure 4. The highest predictive ability (mean) was observed in MS18 (.47; range = .13–.69) followed by OS19 with an average of 0.38 (range = .08–.60). The lowest predictions were observed in the seedling data, which had a mean of .25 and ranged from .01 to .42.

## 4 DISCUSSION

This study is the first to evaluate wheat lines for field response to the *Pgt* race TTRTF. We were successful in identifying 19 (10%) lines with all‐stage resistance to race TTRTF. As new and more virulent *Pgt* races, such as TTRTF, emerge, field screening of germplasm is necessary to identify resistant lines. The single‐race field nursery in Kulumsa was effective for characterizing resistance of wheat germplasm to race TTRTF. Seedling evaluation of Ethiopian common spring wheat germplasm revealed that 56% were resistant as seedings. This proportion of resistant lines is much higher than what was previously reported for North American spring wheat (19.8%; Edae and Rouse (2020)). This finding is consistent with the Ethiopian germplasm being enriched with stem rust resistance genes effective to more virulent races of *Pgt* present in the country (Olivera et al., 2015). Going forward, lines identified with all‐stage or adult‐plant resistance can be used as parents in subsequent wheat breeding by EIAR to mitigate *Pgt* race TTRTF. Our field screening data suggest that race TTRTF is capable of causing high disease responses on a substantial proportion of Ethiopian wheat germplasm. Therefore, screening with race TTRTF would need to be implemented as a part of wheat breeding efforts to select for resistance to this race. As race TTRTF has spread throughout several countries in Africa, Asia, and Europe, screening for resistance to this race should be considered for wheat breeding outside of Ethiopia.

### 4.1 QTL discovery and candidate genes

In this population from the EIAR wheat breeding program, we discovered 20 markers in significant association with TTRTF resistance at seedling and adult‐plant stages. As the QTL discovered in this study explained <10% of the observed disease variation, the QTL could be classified as small to moderate effect. The most consistent QTL were located in 1B (markers *S1B_57131231* and *SCM9* at 57 Mbp and *S1B_175439851* at 175 Mbp) and 2D (marker *S2D_22816678*, 22.3 Mbp). The marker *S1B_175439851* was observed in both Ethiopian field seasons and seedling assays, whereas *S2D_22816678* was observed between the two field seasons, that is, 2018 main season (MS18) and 2019 off season (OS19). Markers *S1B_57131231* and *SCM9*, both located at 57 Mbp (Mihalyov et al., 2017), were significant in the 2019 off season field nursery (OS19) and the seedling assay. The marker *SCM9* is diagnostic for 1BL.1RS rye translocation. In this population, 23 lines have the resistance allele for *S1B_57131231*, of which, 22 also have resistance allele for *SCM9*, the only exception being the line ETBW9562 with low to medium disease scores in both field and seedling data. We therefore postulate that the resistance allele of *S1B_57131231* is correlated with *Sr31* presence and intermediate resistance in this population is provided by *Sr31*. The usability of these consistent markers in marker‐assisted selection to select resistant lines remains to be validated and is an avenue for further research.

The QTL discovered in this study were also positioned closely (within 2 Mbp) to 103 candidate genes from 47 unique gene classes (Supplemental Table S2). Twenty‐seven of the 103 candidate genes belonged to nucleotide‐binding site (NBS) and leucine‐rich repeat (LRR) protein families that are frequently associated with host resistance to pathogens (Chen et al., 2018; Xing et al., 2018). Other notable protein families involved in disease resistance include Ankyrin and WRKY (Wang et al., 2020; Kolodziej et al., 2021), ATP‐binding cassette (ABC) transporters (Krattinger et al., 2019; Banasiak & Jasiński, 2022), and RPM1 and RGA2 (van Wersch et al., 2020). We anticipate that validation of these QTL in future studies will provide additional insight on how the genes may be conferring resistance to TTRTF in both seedling and adult‐plant stages.

### 4.2 Comparison with known genes and QTL

As TTRTF is an emerging *Pgt* race, limited research work has been carried out to genetically characterize plant resistance to this race. In a recently published article, Megerssa et al. (2021) used GWAS in a durum wheat panel to discover nine significant loci in chromosome 6A and two in chromosome 7A conferring seedling resistance to TTRTF. As the markers we discovered in chromosomes 6A and 7A are located more than 600 and 33 Mbp from their markers on respective chromosomes, these regions reported in previous study are likely different from the ones discovered in our study. In a diverse spring wheat panel, Edae and Rouse (2020) found 15 significant loci in chromosomes 1B, 1D, 2A, 2B, and 4D. In our study, we detected two significant markers in 1B—*S1B_57131231* and *S1B_57131236* that are located <4 Mbp away from the marker *S1B_PART1_53250247* reported by Edae and Rouse (2020). Similarly, the marker *S1B_175439851* in this study is ∼5 Mbp away from the marker *S1B_PART1_180406818* reported by Edae and Rouse (2020). While further validation is necessary to establish the relationship among these makers in proximity to each other, it is possible that they are representing the same resistance loci. Additionally, the markers we discovered in chromosomes 2A, 2B, and 4D are >15 Mbp away from the significant markers detected by Edae and Rouse (2020) in those chromosomes and are therefore unlikely to be the same.

We also investigated the colocalization of QTL discovered in this study with (a) genes previously described as effective against TTRTF and (b) adult‐plant resistance genes in wheat that provide a broad‐spectrum resistance to multiple stem rust pathogen races, that is, *Sr2*, *Sr55*, *Sr56*, *Sr57*, and *Sr58*. Currently, genes *Sr24*, *Sr30*, *Sr31*, and *Sr38* are known to be effective against TTRTF (Olivera et al., 2019). *Sr24* and *Sr30* are located on chromosomes 3DL and 5DL, respectively, and we did not detect QTL providing adult‐plant resistance to TTRTF on these chromosomes. We confirmed the presence of *Sr31* in this population, as it was detected during marker screening and GWAS. The gene *Sr38* is located ∼2 Mbp from the marker *S2A_4620729* (4.6 Mbp) in our study and thus could represent the same region (Mourad et al., 2019). As for the known APR genes, no QTL were detected on chromosomes that harbor *Sr2*, *Sr55*, *Sr56*, and *Sr57* (located on 3BS, 4DL, 5B, and 7D, respectively) and thus are not expected to be involved in providing adult‐plant resistance to TTRTF in this population. *Sr58* is located on 1BL and the only 1B marker (*S1B_57131231*) providing field resistance to TTRTF in our study was located on the short arm (57 Mbp; 1B is nearly 690 Mbp long) and thus is unlikely to represent the same gene.

### 4.3 Impact on resistance breeding

One method to improve resistance to stem rust pathogen races is through pyramiding of genes where multiple effective genes are stacked in a set of desirable lines (Bonnett et al., 2005). In particular, a combination of multiple genes and QTL that coexpress could provide lines with near immunity (Joshi & Nayak, 2010). In the two to four QTL combination models explored in this study, we observed up to 13% reduction in disease severity under field conditions and up to 48% at the seedling stage. As the threat of TTRTF persists in the wheat fields of Ethiopia and neighboring nations, stacking a few genes that provide resistance to this race could be crucial in safeguarding the wheat populations and thus should be a priority for the local breeding programs. An ideal strategy for this task is marker‐assisted gene combination followed by field screening of pyramided lines to develop lines with favorable alleles at multiple resistance genes (Zheng et al., 2020; Kumaran et al., 2021).

Another approach to improve disease resistance in a population is the application of GS to increase the frequency of resistant alleles. The effectiveness of GS in boosting stem rust resistance while reducing resource input and breeding timeline has been discussed in previous studies (Haile et al., 2020; Kumar et al., 2021). The usefulness of historical datasets in predicting rust resistance, that is, forward prediction, also has been discussed previously (Rutkoski et al., 2015). Despite a low cost of genotyping on high‐throughput sequencing platforms, we anticipate financial and logistical challenges in genotyping Ethiopian wheat populations each year. In our datasets, mean predictions ranged from .25 (seedling assay) to .47 (main season field nursery). Despite a lack of high predictions, these values suggest the possibility of purging mostly susceptible lines as a viable option, thereby mimicking a truncation selection and increasing the chances of retaining favorable alleles (Isidro et al., 2015). The EIAR wheat breeding program could therefore use the population presented in this study to predict stem rust resistance in future populations. If such an approach were to be adopted, the relationship of future populations with the current (training) population must be evaluated prior to carrying out predictions. This is because a strong relationship between the training population and selection or testing populations is needed to obtain higher prediction accuracies (Zhong et al., 2009).

To summarize, seedling and field studies of *Pgt* race TTRTF confirmed that this emerging race is capable of producing high disease responses on common wheat lines. Integrating screening for resistance to race TTRTF in Ethiopia through seedling and field studies facilitated the identification of resistant breeding lines. Similar approaches could be implemented in other countries where race TTRTF is present. Identification of 20 QTL associated with seedling and field resistance to race TTRTF allows marker‐assisted selection to improve wheat for resistance to race TTRTF. Combinations of specific pairs of QTL reduced TTRTF stem rust response under field conditions. Specific crosses among lines with a higher number of favorable alleles can be used to accumulate alleles associated with resistance to TTRTF. Additionally, GS may be implemented to increase the frequency of resistant alleles in the EIAR wheat breeding germplasm and safeguard the crop against this new, highly virulent stem rust pathogen race.

## AUTHOR CONTRIBUTIONS

Tamrat Negash: Formal analysis; Investigation; Methodology; Resources; Writing‐review & editing. Erena Aka Edae: Investigation; Methodology; Writing‐review & editing. Lidiya Tilahun: Investigation; Methodology; Resources; Writing‐review & editing. James Anderson: Resources; Writing‐review & editing. Matthew N. Rouse: Conceptualization; Data curation; Formal analysis; Funding acquisition; Investigation; Methodology; Project administration; Resources; Supervision; Writing‐original draft; Writing‐review & editing. Prabin Bajgain: Data curation; Formal analysis; Methodology; Software; Visualization; Writing‐original draft; Writing‐review & editing.

## CONFLICT OF INTEREST

The authors declare no conflict of interest.

## Supporting information

Supplemental Table S1: Number of favorable alleles for each line in each dataset.

Supplemental Table S2: Candidate genes discovered within two mega base pairs (2 Mbp) of the significant SNP markers (QTL).

Supplemental Figure S1: Linkage disequilibrium values (*r* ^2^) plotted against physical distance (mega base pairs, Mbp) in the three wheat sub‐genomes. Red line is fitted to display the LD distribution.
