# Supplementary material for: Genome‐wide association mapping for field and seedling resistance to the emerging Puccinia graminis f. sp. tritici race TTRTF in wheat
Source: Plant Genome. 2022 Oct 20;15(4):e20274. doi: 10.1002/tpg2.20274 (PMC12807250; doi:10.1002/tpg2.20274)
Supplement: Supplementary file 1 [file TPG2-15-e20274-s001.docx]

**Supplemental Table S1:** Number of favorable alleles for each line in each dataset.

|  | **MS18** | | **OS19** | | **Seedling** | | **All** |
| --- | --- | --- | --- | --- | --- | --- | --- |
| **Entry** | **No. of favorable alleles** | **No. of significant SNPs** | **No. of favorable alleles** | **No. of significant SNPs** | **No. of favorable alleles** | **No. of significant SNPs** | **Total No. of favorable alleles** |
| Alidoro | 1 | 7 | 6 | 11 | 7 | 8 | 14 |
| Ambera | 2 | 7 | 8 | 11 | 6 | 8 | 16 |
| BW09I113 | 5 | 7 | 8 | 11 | 7 | 8 | 20 |
| BW09I114 | 3 | 7 | 7 | 11 | 2 | 8 | 12 |
| BW09I115 | 6 | 7 | 5 | 11 | 3 | 8 | 14 |
| BW09I116 | 6 | 7 | 7 | 11 | 3 | 8 | 16 |
| BW09I117 | 1 | 7 | 6 | 11 | 7 | 8 | 14 |
| BW09I118 | 5 | 7 | 6 | 11 | 5 | 8 | 16 |
| BW09I119 | 1 | 7 | 4 | 11 | 2 | 8 | 7 |
| BW09I120 | 2 | 7 | 6 | 11 | 5 | 8 | 13 |
| BW09I121 | 3 | 7 | 5 | 11 | 1 | 8 | 9 |
| BW09I122 | 1 | 7 | 6 | 11 | 5 | 8 | 12 |
| BW09I123 | 1 | 7 | 6 | 11 | 7 | 8 | 14 |
| BW09I124 | 6 | 7 | 6 | 11 | 4 | 8 | 16 |
| BWCM267 | 4 | 7 | 6 | 11 | 7 | 8 | 17 |
| BWCM268 | 1 | 7 | 6 | 11 | 7 | 8 | 14 |
| BWCM269 | 2 | 7 | 6 | 11 | 5 | 8 | 13 |
| BWCM270 | 5 | 7 | 9 | 11 | 7 | 8 | 21 |
| BWCM271 | 1 | 7 | 6 | 11 | 6 | 8 | 13 |
| BWCM272 | 4 | 7 | 4 | 11 | 8 | 8 | 16 |
| BWCM273 | 4 | 7 | 7 | 11 | 7 | 8 | 18 |
| BWCM274 | 4 | 7 | 6 | 11 | 8 | 8 | 18 |
| BWCM275 | 1 | 7 | 5 | 11 | 7 | 8 | 13 |
| BWCM276 | 4 | 7 | 7 | 11 | 7 | 8 | 18 |
| BWCM277 | 5 | 7 | 7 | 11 | 7 | 8 | 19 |
| BWIC125 | 3 | 7 | 5 | 11 | 2 | 8 | 10 |
| BWIC126 | 3 | 7 | 5 | 11 | 0 | 8 | 8 |
| BWIC127 | 3 | 7 | 7 | 11 | 2 | 8 | 12 |
| BWIC128 | 2 | 7 | 6 | 11 | 1 | 8 | 9 |
| BWIC129 | 6 | 7 | 6 | 11 | 3 | 8 | 15 |
| BWIC130 | 2 | 7 | 6 | 11 | 6 | 8 | 14 |
| Biqaa | 1 | 7 | 4 | 11 | 4 | 8 | 9 |
| Bulluk | 1 | 7 | 5 | 11 | 8 | 8 | 14 |
| Dashen | 6 | 7 | 5 | 11 | 4 | 8 | 15 |
| Deka | 2 | 7 | 6 | 11 | 6 | 8 | 14 |
|  | **MS18** | | **OS19** | | **Seedling** | | **All** |
| **Entry** | **No. of favorable alleles** | **No. of significant SNPs** | **No. of favorable alleles** | **No. of significant SNPs** | **No. of favorable alleles** | **No. of significant SNPs** | **Total No. of favorable alleles** |
| Dembel | 5 | 7 | 4 | 11 | 7 | 8 | 16 |
| Dereselign | 2 | 7 | 5 | 11 | 6 | 8 | 13 |
| ETBW7956 | 2 | 7 | 5 | 11 | 3 | 8 | 10 |
| ETBW8070 | 5 | 7 | 6 | 11 | 6 | 8 | 17 |
| ETBW8260 | 2 | 7 | 6 | 11 | 8 | 8 | 16 |
| ETBW8261 | 3 | 7 | 6 | 11 | 7 | 8 | 16 |
| ETBW8583 | 4 | 7 | 6 | 11 | 7 | 8 | 17 |
| ETBW8595 | 5 | 7 | 5 | 11 | 6 | 8 | 16 |
| ETBW8668 | 5 | 7 | 6 | 11 | 7 | 8 | 18 |
| ETBW8684 | 1 | 7 | 6 | 11 | 5 | 8 | 12 |
| ETBW8751 | 3 | 7 | 4 | 11 | 5 | 8 | 12 |
| ETBW8804 | 4 | 7 | 5 | 11 | 4 | 8 | 13 |
| ETBW8858 | 1 | 7 | 6 | 11 | 6 | 8 | 13 |
| ETBW8862 | 5 | 7 | 6 | 11 | 7 | 8 | 18 |
| ETBW8870 | 2 | 7 | 4 | 11 | 3 | 8 | 9 |
| ETBW8991 | 5 | 7 | 6 | 11 | 7 | 8 | 18 |
| ETBW9065 | 2 | 7 | 6 | 11 | 8 | 8 | 16 |
| ETBW9066 | 2 | 7 | 6 | 11 | 7 | 8 | 15 |
| ETBW9077 | 5 | 7 | 6 | 11 | 8 | 8 | 19 |
| ETBW9078 | 4 | 7 | 6 | 11 | 8 | 8 | 18 |
| ETBW9080 | 4 | 7 | 6 | 11 | 7 | 8 | 17 |
| ETBW9086 | 4 | 7 | 7 | 11 | 7 | 8 | 18 |
| ETBW9087 | 1 | 7 | 6 | 11 | 7 | 8 | 14 |
| ETBW9089 | 1 | 7 | 6 | 11 | 8 | 8 | 15 |
| ETBW9094 | 5 | 7 | 8 | 11 | 7 | 8 | 20 |
| ETBW9102 | 1 | 7 | 4 | 11 | 5 | 8 | 10 |
| ETBW9109 | 4 | 7 | 6 | 11 | 7 | 8 | 17 |
| ETBW9116 | 1 | 7 | 5 | 11 | 6 | 8 | 12 |
| ETBW9119 | 5 | 7 | 6 | 11 | 8 | 8 | 19 |
| ETBW9128 | 2 | 7 | 3 | 11 | 6 | 8 | 11 |
| ETBW9136 | 5 | 7 | 8 | 11 | 7 | 8 | 20 |
| ETBW9139 | 5 | 7 | 7 | 11 | 7 | 8 | 19 |
| ETBW9149 | 5 | 7 | 7 | 11 | 7 | 8 | 19 |
| ETBW9172 | 4 | 7 | 6 | 11 | 7 | 8 | 17 |
| ETBW9185 | 2 | 7 | 7 | 11 | 5 | 8 | 14 |
| ETBW9193 | 5 | 7 | 5 | 11 | 7 | 8 | 17 |
| ETBW9233 | 2 | 7 | 6 | 11 | 5 | 8 | 13 |
| ETBW9284 | 1 | 7 | 5 | 11 | 6 | 8 | 12 |
|  | **MS18** | | **OS19** | | **Seedling** | | **All** |
| **Entry** | **No. of favorable alleles** | **No. of significant SNPs** | **No. of favorable alleles** | **No. of significant SNPs** | **No. of favorable alleles** | **No. of significant SNPs** | **Total No. of favorable alleles** |
| ETBW9299 | 2 | 7 | 6 | 11 | 6 | 8 | 14 |
| ETBW9304 | 2 | 7 | 6 | 11 | 5 | 8 | 13 |
| ETBW9313 | 5 | 7 | 9 | 11 | 7 | 8 | 21 |
| ETBW9315 | 2 | 7 | 6 | 11 | 7 | 8 | 15 |
| ETBW9396 | 6 | 7 | 5 | 11 | 3 | 8 | 14 |
| ETBW9452 | 1 | 7 | 4 | 11 | 2 | 8 | 7 |
| ETBW9470 | 2 | 7 | 7 | 11 | 5 | 8 | 14 |
| ETBW9486 | 4 | 7 | 6 | 11 | 7 | 8 | 17 |
| ETBW9543 | 6 | 7 | 8 | 11 | 6 | 8 | 20 |
| ETBW9545 | 4 | 7 | 6 | 11 | 7 | 8 | 17 |
| ETBW9547 | 4 | 7 | 8 | 11 | 7 | 8 | 19 |
| ETBW9548 | 1 | 7 | 6 | 11 | 6 | 8 | 13 |
| ETBW9549 | 1 | 7 | 5 | 11 | 5 | 8 | 11 |
| ETBW9550 | 1 | 7 | 5 | 11 | 8 | 8 | 14 |
| ETBW9551 | 4 | 7 | 6 | 11 | 7 | 8 | 17 |
| ETBW9552 | 1 | 7 | 6 | 11 | 7 | 8 | 14 |
| ETBW9553 | 5 | 7 | 6 | 11 | 7 | 8 | 18 |
| ETBW9554 | 4 | 7 | 7 | 11 | 7 | 8 | 18 |
| ETBW9555 | 1 | 7 | 8 | 11 | 7 | 8 | 16 |
| ETBW9556 | 3 | 7 | 5 | 11 | 7 | 8 | 15 |
| ETBW9557 | 2 | 7 | 6 | 11 | 6 | 8 | 14 |
| ETBW9558 | 1 | 7 | 6 | 11 | 5 | 8 | 12 |
| ETBW9559 | 1 | 7 | 6 | 11 | 7 | 8 | 14 |
| ETBW9560 | 2 | 7 | 3 | 11 | 6 | 8 | 11 |
| ETBW9561 | 2 | 7 | 5 | 11 | 7 | 8 | 14 |
| ETBW9562 | 2 | 7 | 7 | 11 | 3 | 8 | 12 |
| ETBW9563 | 5 | 7 | 8 | 11 | 7 | 8 | 20 |
| ETBW9564 | 0 | 7 | 4 | 11 | 7 | 8 | 11 |
| ETBW9565 | 5 | 7 | 6 | 11 | 7 | 8 | 18 |
| ETBW9566 | 2 | 7 | 4 | 11 | 6 | 8 | 12 |
| ETBW9568 | 4 | 7 | 6 | 11 | 7 | 8 | 17 |
| ETBW9569 | 2 | 7 | 5 | 11 | 6 | 8 | 13 |
| ETBW9570 | 2 | 7 | 6 | 11 | 5 | 8 | 13 |
| ETBW9571 | 5 | 7 | 8 | 11 | 6 | 8 | 19 |
| ETBW9572 | 1 | 7 | 5 | 11 | 7 | 8 | 13 |
| ETBW9573 | 4 | 7 | 6 | 11 | 7 | 8 | 17 |
| ETBW9574 | 2 | 7 | 6 | 11 | 6 | 8 | 14 |
| ETBW9575 | 1 | 7 | 6 | 11 | 7 | 8 | 14 |
|  | **MS18** | | **OS19** | | **Seedling** | | **All** |
| **Entry** | **No. of favorable alleles** | **No. of significant SNPs** | **No. of favorable alleles** | **No. of significant SNPs** | **No. of favorable alleles** | **No. of significant SNPs** | **Total No. of favorable alleles** |
| ETBW9576 | 2 | 7 | 4 | 11 | 7 | 8 | 13 |
| ETBW9577 | 5 | 7 | 8 | 11 | 7 | 8 | 20 |
| ETBW9578 | 4 | 7 | 6 | 11 | 7 | 8 | 17 |
| ETBW9579 | 2 | 7 | 6 | 11 | 7 | 8 | 15 |
| ETBW9580 | 1 | 7 | 6 | 11 | 7 | 8 | 14 |
| ETBW9581 | 4 | 7 | 6 | 11 | 7 | 8 | 17 |
| ETBW9582 | 1 | 7 | 6 | 11 | 8 | 8 | 15 |
| ETBW9583 | 4 | 7 | 6 | 11 | 8 | 8 | 18 |
| ETBW9584 | 0 | 7 | 5 | 11 | 8 | 8 | 13 |
| ETBW9585 | 0 | 7 | 5 | 11 | 7 | 8 | 12 |
| ETBW9586 | 4 | 7 | 6 | 11 | 7 | 8 | 17 |
| ETBW9587 | 4 | 7 | 6 | 11 | 7 | 8 | 17 |
| ETBW9588 | 4 | 7 | 6 | 11 | 7 | 8 | 17 |
| ETBW9589 | 2 | 7 | 6 | 11 | 7 | 8 | 15 |
| ETBW9594 | 1 | 7 | 5 | 11 | 7 | 8 | 13 |
| ETBW9597 | 2 | 7 | 6 | 11 | 7 | 8 | 15 |
| ETBW9605 | 1 | 7 | 5 | 11 | 6 | 8 | 12 |
| ETBW9606 | 1 | 7 | 6 | 11 | 7 | 8 | 14 |
| ETBW9611 | 2 | 7 | 6 | 11 | 7 | 8 | 15 |
| ETBW9613 | 1 | 7 | 6 | 11 | 5 | 8 | 12 |
| ETBW9615 | 3 | 7 | 5 | 11 | 4 | 8 | 12 |
| ETBW9616 | 1 | 7 | 6 | 11 | 7 | 8 | 14 |
| ETBW9617 | 5 | 7 | 5 | 11 | 7 | 8 | 17 |
| ETBW9618 | 2 | 7 | 4 | 11 | 4 | 8 | 10 |
| ETBW9619 | 2 | 7 | 5 | 11 | 6 | 8 | 13 |
| ETBW9623 | 3 | 7 | 8 | 11 | 7 | 8 | 18 |
| ETBW9624 | 1 | 7 | 7 | 11 | 6 | 8 | 14 |
| ETBW9626 | 4 | 7 | 6 | 11 | 7 | 8 | 17 |
| ETBW9627 | 1 | 7 | 5 | 11 | 7 | 8 | 13 |
| ETBW9628 | 3 | 7 | 7 | 11 | 7 | 8 | 17 |
| ETBW9635 | 6 | 7 | 6 | 11 | 5 | 8 | 17 |
| ETBW9636 | 1 | 7 | 5 | 11 | 6 | 8 | 12 |
| ETBW9641 | 0 | 7 | 5 | 11 | 8 | 8 | 13 |
| ETBW9642 | 2 | 7 | 7 | 11 | 7 | 8 | 16 |
| ETBW9646 | 5 | 7 | 7 | 11 | 7 | 8 | 19 |
| ETBW9647 | 1 | 7 | 7 | 11 | 6 | 8 | 14 |
| ETBW9648 | 4 | 7 | 6 | 11 | 7 | 8 | 17 |
| ETBW9650 | 5 | 7 | 7 | 11 | 7 | 8 | 19 |
|  | **MS18** | | **OS19** | | **Seedling** | | **All** |
| **Entry** | **No. of favorable alleles** | **No. of significant SNPs** | **No. of favorable alleles** | **No. of significant SNPs** | **No. of favorable alleles** | **No. of significant SNPs** | **Total No. of favorable alleles** |
| ETBW9651 | 1 | 7 | 6 | 11 | 6 | 8 | 13 |
| ETBW9652 | 1 | 7 | 4 | 11 | 7 | 8 | 12 |
| ETBW9653 | 2 | 7 | 5 | 11 | 7 | 8 | 14 |
| ETBW9654 | 5 | 7 | 7 | 11 | 7 | 8 | 19 |
| ETBW9655 | 3 | 7 | 6 | 11 | 7 | 8 | 16 |
| ETBW9657 | 4 | 7 | 6 | 11 | 7 | 8 | 17 |
| ETBW9658 | 4 | 7 | 6 | 11 | 7 | 8 | 17 |
| ETBW9661 | 6 | 7 | 6 | 11 | 2 | 8 | 14 |
| Fentale | 5 | 7 | 5 | 11 | 6 | 8 | 16 |
| Gambo | 5 | 7 | 6 | 11 | 7 | 8 | 18 |
| Hidasse | 2 | 7 | 5 | 11 | 7 | 8 | 14 |
| Hobora | 5 | 7 | 5 | 11 | 8 | 8 | 18 |
| Honkolo | 2 | 7 | 4 | 11 | 7 | 8 | 13 |
| Hulluka | 1 | 7 | 5 | 11 | 7 | 8 | 13 |
| K6295-4A | 0 | 7 | 3 | 11 | 7 | 8 | 10 |
| KBG-01 | 5 | 7 | 8 | 11 | 4 | 8 | 17 |
| Kakaba | 2 | 7 | 5 | 11 | 8 | 8 | 15 |
| Lemu | 5 | 7 | 6 | 11 | 7 | 8 | 18 |
| Liben | 2 | 7 | 7 | 11 | 6 | 8 | 15 |
| Mandoyu | 5 | 7 | 5 | 11 | 7 | 8 | 17 |
| Medewolabu | 6 | 7 | 6 | 11 | 5 | 8 | 17 |
| Monaliza | 2 | 7 | 8 | 11 | 6 | 8 | 16 |
| Ogolcho | 2 | 7 | 5 | 11 | 5 | 8 | 12 |
| Qulqulu | 1 | 7 | 6 | 11 | 6 | 8 | 13 |
| Sanate | 3 | 7 | 4 | 11 | 6 | 8 | 13 |
| Shorima | 2 | 7 | 6 | 11 | 7 | 8 | 15 |
| Sofumer | 2 | 7 | 2 | 11 | 3 | 8 | 7 |
| Tay | 6 | 7 | 5 | 11 | 3 | 8 | 14 |
| Wane | 5 | 7 | 8 | 11 | 7 | 8 | 20 |
