# Supplementary material for: Genome‐wide association mapping for field and seedling resistance to the emerging Puccinia graminis f. sp. tritici race TTRTF in wheat
Source: Plant Genome. 2022 Oct 20;15(4):e20274. doi: 10.1002/tpg2.20274 (PMC12807250; doi:10.1002/tpg2.20274)
Supplement: Supplementary file 2 [file TPG2-15-e20274-s002.docx]

**Supplemental Table S2:** Candidate genes discovered within two mega base pairs (2 Mbp) of the significant SNP markers (QTL).

| **QTL** | **Chromosome** | **Gene ID** | **Gene Description** | **Pfam IDs** |
| --- | --- | --- | --- | --- |
| S1A_57399833 | 1A | TraesCS1A01G073200.1 | Leucine-rich repeat receptor-like protein kinase family protein | PF08263; PF13855; PF00560 |
| S1A_57399833 | 1A | TraesCS1A01G073300.1 | Kinase, putative | PF01657; PF07714 |
| S1A_575239272 | 1A | TraesCS1A01G415300.1 | Mitogen-activated protein kinase | PF00069 |
| S1A_575239272 | 1A | TraesCS1A01G416200.1 | Kinase family protein | PF07714 |
| S1A_575239272 | 1A | TraesCS1A01G416400.1 | Leucine-rich repeat (LRR) family protein | NA |
| S1A_575239272 | 1A | TraesCS1A01G417000.1 | Disease resistance protein RPM1 | PF00931 |
| S1A_575239272 | 1A | TraesCS1A01G417600.1 | S-locus lectin protein kinase family protein | NA |
| S1A_575239272 | 1A | TraesCS1A01G418700.2 | Receptor-like protein kinase | PF08263; PF13855; PF00069 |
| S1B_57131231; S1B_57131236 | 1B | TraesCS1B01G073400.1 | S-locus lectin protein kinase family protein | NA |
| S1B_175439851 | 1B | TraesCS1B01G137500.1 | Protein kinase-like | PF00069 |
| S1B_175439851 | 1B | TraesCS1B01G137600.1 | Glycosyltransferase | PF00201 |
| S1B_489545605 | 1B | TraesCS1B01G281100.1 | Kinase family protein | PF00069 |
| S2A_4620729 | 2A | TraesCS2A01G008400.1 | Homeobox leucine zipper protein | NA |
| S2A_4620729 | 2A | TraesCS2A01G008500.1 | NBS-LRR-like resistance protein | NA |
| S2A_4620729 | 2A | TraesCS2A01G008600.1 | Disease resistance protein RGA2 | NA |
| S2A_4620729 | 2A | TraesCS2A01G008700.1 | NBS-LRR-like resistance protein | NA |
| S2A_4620729 | 2A | TraesCS2A01G008800.1 | NBS-LRR-like resistance protein | NA |
| S2A_4620729 | 2A | TraesCS2A01G008900.1 | NBS-LRR-like resistance protein | PF00931 |
| S2A_4620729 | 2A | TraesCS2A01G009500.1 | Glycosyltransferase | PF00201 |
| S2A_4620729 | 2A | TraesCS2A01G009600.1 | ABC transporter B family-like protein | PF00664; PF00005 |
| S2A_4620729 | 2A | TraesCS2A01G009700.1 | ABC transporter B family-like protein | PF00664; PF00005 |
| S2A_4620729 | 2A | TraesCS2A01G009900.1 | ABC transporter B family-like protein | PF00664; PF00005 |
| S2A_4620729 | 2A | TraesCS2A01G010300.1 | Receptor protein kinase, putative | PF08263; PF13855; PF00069 |
| S2A_4620729 | 2A | TraesCS2A01G010400.1 | Leucine-rich repeat receptor-like protein kinase family protein, putative | PF13855; PF12799 |
| S2A_4620729 | 2A | TraesCS2A01G010500.1 | Leucine-rich repeat receptor-like kinase | PF08263; PF13855 |
| S2A_4620729 | 2A | TraesCS2A01G011000.1 | WRKY transcription factor | PF03106 |
| S2A_4620729 | 2A | TraesCS2A01G012400.1 | Leucine-rich repeat receptor-like protein kinase family protein | PF13855; PF00560 |
| S2A_4620729 | 2A | TraesCS2A01G012500.1 | Receptor-kinase, putative | PF08263; PF00560; PF13855; PF00069 |
| S2A_4620729 | 2A | TraesCS2A01G012900.1 | Receptor protein kinase | PF13855; PF00069 |
| S2B_67087867 | 2B | TraesCS2B01G105800.1 | Receptor-like protein kinase | PF00560; PF00069 |
| S2B_67087867 | 2B | TraesCS2B01G106100.1 | Lectin receptor kinase | PF00069 |
| S2B_67087867 | 2B | TraesCS2B01G106200.1 | Lectin receptor kinase | PF00139; PF00069 |
| S2B_67087867 | 2B | TraesCS2B01G106900.1 | Receptor-like kinase | PF13855; PF07714 |
| S2D_22816678; S2D_22816689; S2D_22816672; S2D_22816702 | 2D | TraesCS2D01G054800.1 | Glycosyltransferase | PF00201 |
| S2D_22816678; S2D_22816689; S2D_22816672; S2D_22816702 | 2D | TraesCS2D01G054900.2 | Glycosyltransferase | PF00201 |
| S2D_22816678; S2D_22816689; S2D_22816672; S2D_22816702 | 2D | TraesCS2D01G055000.1 | Glycosyltransferase | PF00201 |
| S2D_22816678; S2D_22816689; S2D_22816672; S2D_22816702 | 2D | TraesCS2D01G055100.1 | Glycosyltransferase | PF00201 |
| S2D_22816678; S2D_22816689; S2D_22816672; S2D_22816702 | 2D | TraesCS2D01G055200.1 | Glycosyltransferase | NA |
| S2D_22816678; S2D_22816689; S2D_22816672; S2D_22816702 | 2D | TraesCS2D01G055600.1 | ABC subfamily C transporter | PF00664; PF00005 |
| S2D_22816678; S2D_22816689; S2D_22816672; S2D_22816702 | 2D | TraesCS2D01G055800.1 | ABC subfamily C transporter | PF00664; PF00005 |
| S2D_22816678; S2D_22816689; S2D_22816672; S2D_22816702 | 2D | TraesCS2D01G055900.1 | Protein kinase | PF00139; PF07714 |
| S2D_22816678; S2D_22816689; S2D_22816672; S2D_22816702 | 2D | TraesCS2D01G056300.2 | Multidrug resistance protein ABC transporter family protein | PF00664; PF00005 |
| S2D_22816678; S2D_22816689; S2D_22816672; S2D_22816702 | 2D | TraesCS2D01G056600.1 | Leucine-rich repeat receptor-like protein kinase family protein | PF08263; PF00560; PF13855; PF13516 |
| S2D_22816678; S2D_22816689; S2D_22816672; S2D_22816702 | 2D | TraesCS2D01G056800.1 | Disease resistance protein (TIR-NBS-LRR class) family | NA |
| S2D_22816678; S2D_22816689; S2D_22816672; S2D_22816702 | 2D | TraesCS2D01G057300.1 | Leucine-rich repeat receptor-like protein kinase family protein | PF08263; PF00560; PF13855 |
| S2D_22816678; S2D_22816689; S2D_22816672; S2D_22816702 | 2D | TraesCS2D01G057600.1 | disease resistance protein (TIR-NBS-LRR class) | PF00931; PF01419 |
| S2D_22816678; S2D_22816689; S2D_22816672; S2D_22816702 | 2D | TraesCS2D01G057700.1 | receptor kinase 1 | PF00069; PF00635 |
| S2D_591388465 | 2D | TraesCS2D01G493700.1 | Protein kinase | PF00069 |
| S3D_931530 | 3D | TraesCS3D01G000900.1 | Leucine-rich repeat protein kinase family protein | NA |
| S3D_931530 | 3D | TraesCS3D01G001000.1 | Disease resistance protein RPM1 | NA |
| S3D_931530 | 3D | TraesCS3D01G001100.1 | Disease resistance protein | PF00931 |
| S3D_931530 | 3D | TraesCS3D01G001400.1 | Glycosyltransferase | PF00201 |
| S3D_931530 | 3D | TraesCS3D01G001500.1 | Dephospho-CoA kinase, putative, expressed | PF01121 |
| S3D_931530 | 3D | TraesCS3D01G001700.1 | Disease resistance protein | PF00931 |
| S3D_931530 | 3D | TraesCS3D01G002100.1 | Receptor kinase-like protein | PF00069 |
| S3D_931530 | 3D | TraesCS3D01G002200.1 | Protein kinase, putative | PF07714 |
| S3D_931530 | 3D | TraesCS3D01G002400.2 | disease resistance protein (TIR-NBS-LRR class) | PF00931 |
| S3D_931530 | 3D | TraesCS3D01G002500.1 | Disease resistance protein (NBS-LRR class) family | NA |
| S3D_931530 | 3D | TraesCS3D01G002700.1 | Disease resistance protein RPM1 | PF00931; PF00085 |
| S3D_931530 | 3D | TraesCS3D01G002800.1 | Disease resistance protein (NBS-LRR class) family | PF00931 |
| S3D_931530 | 3D | TraesCS3D01G003400.1 | Wall-associated receptor kinase 5 | NA |
| S3D_931530 | 3D | TraesCS3D01G003700.1 | Receptor-kinase, putative | PF13855; PF00069 |
| S3D_931530 | 3D | TraesCS3D01G003900.1 | Protein kinase family protein | PF13947; PF07645; PF00069 |
| S4D_18464711 | 4D | TraesCS4D01G041600.1 | DNA-binding storekeeper protein-related transcriptional regulator | PF04504 |
| S6A_1757444 | 6A | TraesCS6A01G001300.1 | disease resistance protein (TIR-NBS-LRR class) | NA |
| S6A_1757444 | 6A | TraesCS6A01G001700.2 | Receptor-like kinase | PF13947; PF00069 |
| S6A_1757444 | 6A | TraesCS6A01G001800.1 | Protein kinase, putative | PF12819; PF12799; PF07714 |
| S6A_1757444 | 6A | TraesCS6A01G001900.1 | Protein kinase, putative | PF12819; PF12799; PF07714 |
| S6A_1757444 | 6A | TraesCS6A01G003800.1 | NAC domain protein | PF02365 |
| S6A_1757444 | 6A | TraesCS6A01G004100.1 | Receptor-kinase, putative | PF08263; PF00560; PF13855; PF00069 |
| S6A_1757444 | 6A | TraesCS6A01G004500.1 | Receptor-like protein kinase | PF00069; PF00635 |
| S6A_1757444 | 6A | TraesCS6A01G004600.1 | Ankyrin repeat family protein | PF13637 |
| S6A_1757444 | 6A | TraesCS6A01G005000.1 | Protein ENHANCED DISEASE RESISTANCE 2-like | PF01852; PF07059 |
| S6A_1757444 | 6A | TraesCS6A01G005100.1 | Pathogen-related protein | NA |
| S6D_2750348 | 6D | TraesCS6D01G003800.1 | Protein kinase, putative | PF12819; PF12799; PF07714 |
| S6D_2750348 | 6D | TraesCS6D01G003900.3 | Receptor-like protein kinase | PF13947; PF00069 |
| S6D_2750348 | 6D | TraesCS6D01G004000.1 | Protein kinase, putative | PF12819; PF12799; PF07714 |
| S6D_2750348 | 6D | TraesCS6D01G005300.1 | NBS-LRR-like resistance protein | PF00931 |
| S6D_2750348 | 6D | TraesCS6D01G005700.1 | casein kinase I | NA |
| S6D_2750348 | 6D | TraesCS6D01G005800.1 | Leucine-rich repeat receptor-like protein kinase family protein | PF08263; PF13516; PF00560; PF13855 |
| S6D_2750348 | 6D | TraesCS6D01G005900.1 | Leucine-rich repeat receptor-like protein kinase family protein | PF08263; PF00560 |
| S6D_2750348 | 6D | TraesCS6D01G006000.1 | Leucine-rich repeat receptor-like protein kinase family protein | PF00560; PF13855 |
| S6D_2750348 | 6D | TraesCS6D01G006600.1 | Ankyrin repeat family protein | PF13637 |
| S6D_2750348 | 6D | TraesCS6D01G007400.1 | Pathogen-related protein | NA |
| S6D_2750348 | 6D | TraesCS6D01G007500.1 | Glycosyltransferase | PF00201 |
| S6D_2750348 | 6D | TraesCS6D01G007600.1 | Protein ENHANCED DISEASE RESISTANCE 2-like | PF01852; PF07059 |
| S6D_2750348 | 6D | TraesCS6D01G007800.1 | receptor kinase 1 | PF00069 |
| S6D_2750348 | 6D | TraesCS6D01G007900.1 | disease resistance protein (TIR-NBS-LRR class) | NA |
| S6D_2750348 | 6D | TraesCS6D01G009200.1 | Serine/threonine-protein kinase WNK1 | NA |
| S6D_2750348 | 6D | TraesCS6D01G009400.1 | Ankyrin repeat domain-containing protein CP77 | NA |
| S7A_733820033 | 7A | TraesCS7A01G561900.4 | NBS-LRR disease resistance protein | PF00931 |
| S7A_733820033 | 7A | TraesCS7A01G562000.1 | NBS-LRR disease resistance protein | PF00931 |
| S7A_733820033 | 7A | TraesCS7A01G562400.1 | Disease resistance protein RPM1 | PF00931 |
| S7A_733820033 | 7A | TraesCS7A01G562500.1 | NBS-LRR disease resistance protein | PF00931 |
| S7A_733820033 | 7A | TraesCS7A01G562800.1 | Disease resistance protein RPM1 | PF00931 |
| S7A_733820033 | 7A | TraesCS7A01G562900.2 | Disease resistance protein RPM1 | PF00931 |
| S7A_733820033 | 7A | TraesCS7A01G563100.2 | Receptor-like protein kinase | PF13947; PF07714 |
| S7A_733820033 | 7A | TraesCS7A01G563300.1 | Pathogenesis-related (PR)-10-related norcoclaurine synthase-like protein | PF00407 |
| S7A_733820033 | 7A | TraesCS7A01G564000.1 | Disease resistance protein (NBS-LRR class) family | PF00931; PF13855 |
| S7A_733820033 | 7A | TraesCS7A01G564900.2 | receptor kinase 1 | PF00069; PF00400 |
| S7A_733820033 | 7A | TraesCS7A01G565100.1 | Pathogenesis-related protein 1 | PF00188 |
| S7A_733820033 | 7A | TraesCS7A01G565200.1 | Receptor-like protein kinase | PF13947; PF07645; PF00069 |
| S7B_125884505 | 7B | TraesCS7B01G110700.1 | Receptor-like protein kinase | PF08263; PF00560; PF00069 |
