# Supplementary material for: Genome‐wide association mapping for field and seedling resistance to the emerging Puccinia graminis f. sp. tritici race TTRTF in wheat
Source: Plant Genome. 2022 Oct 20;15(4):e20274. doi: 10.1002/tpg2.20274 (PMC12807250; doi:10.1002/tpg2.20274)
Supplement: Supplementary file 3 [file TPG2-15-e20274-s003.docx]

**Supplemental Figure S1:** Linkage disequilibrium values (*r^2^*) plotted against physical distance (mega base pairs, Mbp) in the three wheat sub-genomes. Red line is fitted to display the LD distribution.
